# A Case of Presumed Tuberculosis Uveitis with Occlusive Vasculitis from an Endemic Region

**DOI:** 10.4274/tjo.32548

**Published:** 2017-06-01

**Authors:** Berna Başarır, Yalçın Karaküçük, Çiğdem Altan, Banu Şatana, Bulut Ocak, Aslı İnal

**Affiliations:** 1 Beyoğlu Eye Training and Research Hospital, Ophthalmology Clinic, İstanbul, Turkey

**Keywords:** Tuberculosis, Uveitis, endemic area

## Abstract

In this report, we present a case with presumed unilateral tuberculosis uveitis from an endemic region. A 23-year-old male presented with decreased vision in his left eye for 15 days. Visual acuities were 1.0 in his right eye and 0.3 in his left eye. Ophthalmologic examination was normal for the right eye. Slit-lamp examination revealed 2+ cells in the vitreous without anterior chamber reaction in his left eye. Fundus examination revealed occlusive vasculitis and granuloma. His history revealed that he had a respiratory infection with fever 3 months ago while visiting his native country, Rwanda, and was treated with non-specific antibiotic therapy. His visual symptom started 2 weeks after his systemic symptoms resolved. Laboratory findings included 15 mm induration in purified protein derivative tuberculin skin test, HIV negativity, and parenchymal lesions in chest X-ray. Bronchoalveolar lavage was negative for acid-fast bacillus. A pulmonary disease consultant reported presumed tuberculosis because of the patient’s history. Anti-tuberculosis treatment was initiated. The patient’s visual acuity improved rapidly and his signs regressed. A careful history should be taken from patients with uveitis. Travel to tuberculosis-endemic areas may be important for diagnosis and should be asked about directly.

## INTRODUCTION

Tuberculosis (TB) is a chronic granulomatous disease caused by the *Mycobacterium* family, which are aerobic, intracellular/acid-fast staining, non-spore-forming, nonmotile bacilli. In humans, the agents responsible for TB are M. tuberculosis, transmitted via aerosol droplets, and *M. bovis*, transmitted through unpasteurized milk. Other atypical mycobacteria, such as *M. avium* complex, may also cause disease in immunodeficient individuals.^1^

Approximately one-third of the global population is infected with TB bacilli. Although 33% of these cases are in southeast Asia, the highest mortality rates occur in Africa due to the high prevalence of HIV.^2^ TB primarily affects the lungs due to the inhalation of infectious droplets (primary TB), and in 80% of cases, the disease is limited in the lungs by cellular immunity and is asymptomatic (latent TB). Conditions which affect cellular immunity may lead to infection through the activation of latent bacteria (post-primary TB). The bacteria may also spread through lymphatic and hematogenic pathways to involve extrapulmonary tissues such as the gastrointestinal system, genitourinary system, cardiovascular system, skin, central nervous system, and eye. These tissues may be affected in isolation or simultaneously with the pulmonary system.^3^

It is estimated that 1.4% of patients with pulmonary TB develop ocular signs.^4^ Conversely, pulmonary TB is not seen in the majority of patients with ocular TB.^5,6^ The ocular system is affected in nearly 20% of extrapulmonary TB patients.^2^ In addition to involvement of the eyelids, conjunctiva, cornea, sclera, extraocular muscles, optic nerve, and orbit, there may also be intraocular involvement.

Intraocular TB can manifest as a wide clinical spectrum including granulomatous anterior uveitis, chronic anterior uveitis, intermediate uveitis, retinal vasculitis, serpiginous-like choroiditis, choroidal granuloma, neuroretinitis, and panuveitis.^7,8^

In this report, we present a case from an endemic region diagnosed as presumed TB whose uveitis completely resolved with antituberculous therapy.

## CASE REPORT

A 23-year-old male patient presented to our clinic with complaints of decreased vision in his left eye for 15 days. We learned from his history that he was a native of Rwanda and while visiting there about 3 months earlier, he had a respiratory infection with fever and rash and had been treated for 1.5 months with empiric antibiotic therapy (doxycycline + cephalosporin), during which his respiratory infection had resolved.

**Figure 1:**
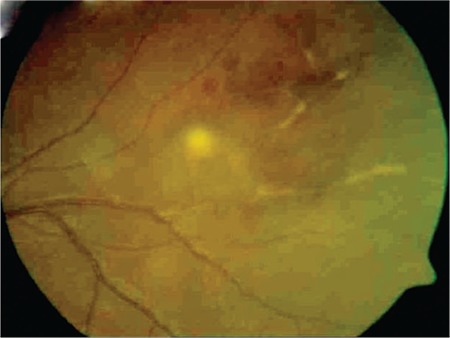
Occlusive vasculitis and choroiditis focus in the left eye

**Figure 2:**
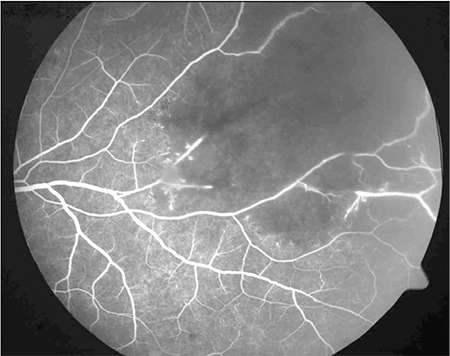
Left eye fluorescein angiography showing superotemporal areas of ischemia due to occlusive vasculitis

**Figure 3:**
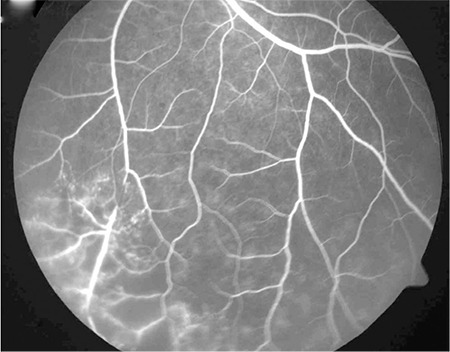
Left eye fluorescein angiography showing inferior areas of ischemia

On ophthalmologic examination, his visual acuity was 1.0 (Snellen chart) in the right eye and 0.3 in the left. Slit-lamp examination of the right eye was normal, intraocular pressure was 9 mmHg, the vitreous was clear and the fundus appeared normal. In the left eye, slit-lamp examination revealed pigmented granulomatous keratic precipitates in the corneal endothelium and iris pigments on the lens. Intraocular pressure was 6 mmHg, and 2+ vitreous cells were observed; fundus examination revealed widespread occlusive vasculitis foci, retinal hemorrhages in the inferior and temporal quadrants, snowball opacities in the inferior, and a focus of choroiditis at 2 o’clock (Figure 1). Fundus fluorescein angiography (FA) was normal in the right eye. In the left eye, FA revealed dye leakage at the optic nerve head and superotemporal branch vein, a choroiditis focus in the inferotemporal vascular arcade showing early hypofluorescence and late hyperfluorescence with leakage, and hypofluorescence due to ischemia and vascular leakage in the temporal periphery (Figures 2 and 3).

Evaluated with clinical examination findings, the lesions in the left eye were considered to be papillitis, occlusive vasculitis, and choroiditis. In laboratory tests, widespread stromal infiltration was observed in the right lung and the left lung was normal on posterior-anterior chest X-ray; purified protein derivative (PPD) tuberculin skin test resulted in a 15 mm induration (the patient had no TB vaccination scar); whole blood and biochemical values were normal; hepatitis serology was negative; HIV ELISA test was negative, serum angiotensin converting enzyme level was 73 U/L, serum calcium level was normal, Brucella agglutination test was negative, and syphilis serology was negative. In light of these clinical and laboratory findings, the patient was diagnosed as suspected TB uveitis and a consultation with the department of pulmonary diseases was requested.

Bronchial fluid was negative in acid-fast bacillus staining. Bronchoscopy revealed mucosal swelling in the bronchial mucosa of the right upper lobe and the left main bronchus near its bifurcation. Biopsy results from these areas of swelling indicated granulomatous inflammatory tissue. After consultation with a pulmonologist, a 4-drug anti-TB regimen (isoniazid, rifampicin, pyrazinamide, ethambutol) was initiated. After 1 month of treatment, the patient’s visual acuity had improved to 0.8 and the vasculitis and choroiditis foci had regressed. Additionally, argon laser scatter photocoagulation was applied to the ischemic area in the left eye. In consultation with the department of pulmonary diseases, regression of the pulmonary lesions was observed and treatment was changed to a 2-drug anti-TB regimen (isoniazid and rifampicin).

**Figure 4:**
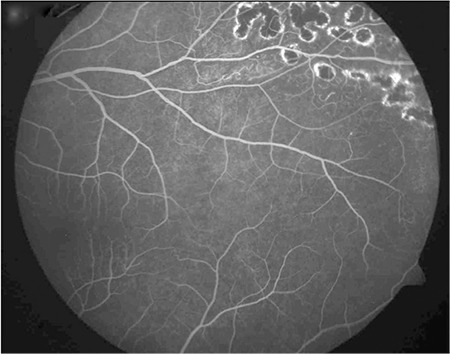
Left eye fluorescein angiography showing scars from laser photocoagulation applied to the areas of ischemia

**Figure 5:**
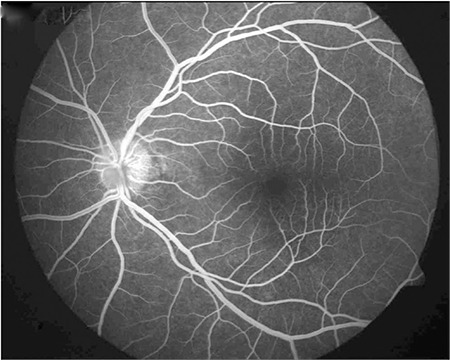
The posterior pole of the left eye appears normal on fluorescein angiography performed after 9 months of treatment

In month 9 of anti-TB therapy, visual acuity was 1.0 in the right eye, intraocular pressure was 10 mmHg, and slit-lamp and fundus examinations were normal. In the left eye, visual acuity was 1.0, intraocular pressure was 9 mmHg, iris pigments on the lens were observed on slit-lamp examination, and argon laser photocoagulation scars were apparent in the temporal and inferior periphery on fundus examination. On FA, photocoagulation scars corresponding to the ischemic areas were visible and the optic nerve leakage had resolved (Figures 4 and 5). Treatment was discontinued after completing a total of 9 months of anti-TB therapy. No recurrence was observed during 2 years of follow-up.

## DISCUSSION

Eighty percent of the total global TB is found in 22 countries: India, China, Indonesia, Bangladesh, Pakistan, Nigeria, the Philippines, South Africa, the Russian Federation, Ethiopia, Vietnam, the Democratic Republic of the Congo, Brazil, Tanzania, Kenya, Thailand, Myanmar, Afghanistan, Uganda, Peru, Zimbabwe, and Cambodia.^9^ Drug and alcohol use, low sociocultural status, general ethnic differences in health status, and differences in access to health care are accepted as factors that may explain the unequal global distribution of the disease.^10^ In developing countries, TB is the most common opportunistic infection in HIV-infected individuals due to poor hygiene, lack of sanitation, poverty, and drug resistance.^9^ An epidemiologic study conducted in tertiary health centers in our country Turkey determined TB as the etiologic agent in 0.3% of uveitis patients.^11^

In the absence of histopathologic or microbiologic findings, there is no gold standard diagnostic method for TB uveitis.^6^ Ocular TB is a wide-spectrum clinical entity that is difficult to diagnose and requires the expertise of both pulmonologists and ophthalmologists for treatment and follow-up.^6,8^ It typically manifests with granulomatous anterior uveitis (nongranulomatous inflammation is rare) with or without iris nodules; intermediate uveitis; ciliary body tuberculoma; posterior uveitis, often in the form of choroidal tubercle or tuberculoma; retinal vasculitis (particularly venous); vitritis; retinal hemorrhages; neovascularization; serpiginous-like choroiditis; and rarely, neuroretinitis, endophthalmitis, or panophthalmitis.^5,8,9^ In a study investigating ocular signs predictive of TB uveitis, Gupta et al.^8^ found that the presence of broad-based posterior synechia, retinal vasculitis with or without choroiditis, and serpiginous-like choroiditis were strong indicators of TB uveitis in TB-endemic areas. Choroidal lesions have also been reported as the most common sign in patients with confirmed ocular TB diagnosis.^12^ Our patient exhibited vitritis, choroiditis, and occlusive vasculitis. Although the patient’s lesions regressed with anti-TB therapy, we also performed argon laser photocoagulation on the ischemic areas of the retina because the patient came from abroad and may not have been able to attend follow-up. Because our case was from a TB-endemic area and had a history of pulmonary infection, ocular TB was suspected and treatment was initiated accordingly, despite not being able to confirm the diagnosis with bronchoalveolar lavage culture or histopathologic analysis.

Our patient’s native country Rwanda is endemic for both TB and acquired AIDS. HIV has increased the incidence of TB in Africa (sub-Saharan), which has the highest TB-related mortality rate worldwide.^2^ Therefore, HIV testing was performed immediately in our patient and was negative. However, it should be kept in mind that HIV positivity may cause anergy to the PPD test and make TB diagnosis difficult.

FA is the most commonly ocular imaging method used in the diagnosis of intraocular TB. Tubercles show hypofluorescence in the early phase and hyperfluorescence in the late phase. Retinal vasculitis appears as fluorescein leakage, particularly from the retinal veins. Imaging of the peripheral retina is important for photocoagulation of peripheral capillary nonperfusion and accompanying neovascularization. Indocyanine green angiography is another imaging modality that is useful for measuring and evaluating choroidal involvement and monitoring treatment response in tuberculous posterior uveitis.^13^ Optical coherence tomography (OCT) complements fundus photography and FA in uveitis patients. OCT facilitates the visualization of cystic macular edema and subretinal membranes. It is effective in determining visual prognosis and can also be used to evaluate treatment response. Spectral domain OCT allows choroidal imaging in patients with intraocular inflammation.^14^ Recently, measuring the thickness of the choroid and its individual layers has become possible with enhanced depth imaging (EDI)-OCT technology. Mehta et al.^15^ reported increased choroidal thickness in TB-related active granulomatous uveitis and stated that EDI-OCT may be useful in the diagnosis and follow-up of the disease. Ultrasonography may facilitate the differentiation of tuberculomas from malignant masses. Ultrasound biomicroscopy can assist the visualization of pars plana granulomas in eyes with seclusio pupillae or cataract.^16^

Proof positive tests for intraocular TB include demonstrating the presence of TB bacilli in secretion, fluid, or affected tissue specimens by acid-fast staining, culture, or amplification of bacterial nucleic acids. PCR is a highly sensitive and specific diagnostic test that replicates mycobacterial DNA. This method is especially advantageous when analyzing intraocular fluid because it requires a very small sample volume.^9^ The PPD tuberculin skin test and interferon gamma release assay (QuantiFERON-TB Gold test, T SPOT TB test) assist diagnosis of latent TB. Pulmonary radiographic and tomographic imaging are other auxiliary modalities in intraocular TB.

Diagnostic criteria for intraocular TB have been developed based on laboratory results, clinical parameters, follow-up examinations, and response to anti-TB therapy.^16^ According to these criteria, the presence of clinical signs together microscopic evidence of acid-fast bacilli or positive *M. tuberculosis* culture from ocular fluid is accepted as confirmed intraocular TB. Clinical signs together with positive PPD test, the presence of previous or active TB lesions on pulmonary X-ray, confirmed extrapulmonary TB (by microscopy or *M. tuberculosis* culture of the affected area), or the exclusion of other causes of uveitis, and positive response to a 4-drug anti-TB regimen within a period of 4-6 weeks is considered presumed intraocular TB. It is recommended that anti-TB therapy be initiated and monitored by a physician with expertise in TB. Ocular response to anti-TB therapy should be evaluated by an ophthalmologist.^16^

The treatment recommended for ocular TB is the same as that recommended for pulmonary involvement, and should be adjusted according to the patient’s immune status. The main drugs utilized in therapy are isoniazid, rifampicin, ethambutol, and pyrazinamide. For patients with compromised immunity or disseminated TB, extending the 2-drug regimen to 7 months (total 9 months) is recommended.^17^ When treating ocular TB, corticosteroids should be initiated with anti-TB therapy to reduce the tissue damage that may result from delayed hypersensitivity reaction and control inflammation. Corticosteroid dosage should be reduced gradually based on clinical response and discontinued after 4-6 weeks. However, the use of steroids alone, without anti-TB therapy, should be strictly avoided.^16^ We were able to control uveitis in our patient with anti-TB therapy alone, and did not add steroid therapy.

This case report highlights the importance of early diagnosis and treatment to avoid ocular complications in TB uveitis. We excluded other uveitis etiologies in our patient and initiated anti-TB therapy with a diagnosis of presumed TB uveitis, and treatment was successful. The fact that the patient came from a TB-endemic area immediately suggested a diagnosis of TB.

## References

[1] Brooks GF, Carrol KC, Butel JS, Morse SA, Mietzner TA (2013). Jawetz Melnick & Adelberg’s Medical Microbiology, 26th Edition, USA. Mc Graw Hill LANGE.

[2] Gupta A, Gupta V, Bansal R, Arora S, Pradeep B (2009). Ocular Tuberculosis, In; Gupta A, Gupta V, Herbort CP, Khairallah M eds. veitis Text and Imaging (First edition). UJaypee Brothers Medical Publishers.

[3] Glassroth J, Robins AG, Snider DE (1980). Tuberculosis in the 1980s. N Engl J Med..

[4] Donahue HC (1967). Ophthalmologic experience in a tuberculosis sanatorium. Am J Ophthalmol..

[5] Önal S, Tuğal-Tutkun İ (2011). Ocular Tuberculosis I: Epidemiology, Pathogenesis and Clinical Features. Turk J Ophthalmol..

[6] Gupta A, Gupta V (2005). Tubercular posterior uveitis. Int Ophthalmol Clin..

[7] Akduman L, Aydın O’dwyer P (2008). Oküler Tüberküloz, Bölüm 4, Kısım 4, Üveit el kitabı, Tunç M. Güneş Tıp Kitabevleri..

[8] Gupta A, Bansal R, Gupta V, Sharma A, Bambery P (2010). Ocular signs predictive of tubercular uveitis. Am J Ophthalmol..

[9] Gupta V, Gupta A, Rao NA (2007). Intraocular tuberculosis-an update. Surv Ophthalmol..

[10] Centers for Disease Control and Prevention (CDC) (2004). Racial disparities in Tuberculosis-selected southeastern states, 1991-2002. MMWR Morb Mortal Wkly Rep..

[11] Kazokoglu H, Onal S, Tugal-Tutkun I, Mirza E, Akova Y, Ozyazgan Y, Soylu M, Batioglu F, Apaydin C (2008). Demographic and clinical features of uveitis in tertiary centers in Turkey. Ophthalmic Epidemiol..

[12] Sheu SJ, Shyu JS, Chen LM, Chen YY, Chirn SC, Wang JS (2001). Ocular manifestations of tuberculosis. Ophthalmology..

[13] Wolfensberger TJ, Piguet B, Herbort CP (1999). Indocyanine green angiographic features in tuberculous chorioretinitis. Am J Ophthalmol..

[14] Fung AT, Kaliki S, Shields CL, Mashayekhi A, Shields JA (2013). Solitary idiopathic choroiditis: findings on enhanced depth imaging optical coherence tomography in 10 cases. Ophthalmology..

[15] Mehta H, Sim DA, Keane PA, Zarranz-Ventura J, Gallagher K, Egan CA, Westcott M, Lee RW, Tufail A, Pavesio CE (2015). Structural changes of the choroid in sarcoid- and tuberculosis-related granulomatous uveitis. Eye (Lond)..

[16] Önal S, Tuğal-Tutkun İ (2011). Ocular Tuberculosis II: Diagnosis and treatment. Turk J Ophthalmol..

[17] Centers for Disease Control and Prevention (CDC), American Thoracic Society (2003). Update: adverse event data and revised American Thoracic Society/CDC recommendations against the use of rifampin and pyrazinamide for treatment of latent tuberculosis infection-United States, 2003. MMWR Morb Mortal Wkly Rep..

